# Delirious Mania in an Elderly, Challenges in Diagnosis and Treatment

**DOI:** 10.1155/2023/8984062

**Published:** 2023-11-16

**Authors:** Omkar Dhungel, Najina Shrestha, Pawan Sharma, Pankaj Pathak, Nidesh Sapkota

**Affiliations:** Department of Psychiatry, Patan Academy of Health Sciences, School of Medicine, Lalitpur, Nepal

## Abstract

Delirious mania is an acute neurobehavioral syndrome which can have the features of mania, delirium, psychosis and catatonia. There are no diagnostic and treatment guidelines of delirious mania which can lead to delayed treatment, increasing morbidity and mortality. The primary goal of this report is to raise awareness among healthcare professionals and improve patient outcomes for this potentially life-threatening condition. In this case report, we present an octogenarian female, a case of bipolar disorder, current episode manic, who had impaired orientation, delusion of persecution, and altered sleep–wake cycle. She was treated with a combination of mood stabilizer and antipsychotic and discharged after 24 days of admission.

## 1. Introduction

Delirium is generally considered on the background of medical illness and mania is a psychiatric illness mostly with no medical cause. The term “delirious mania” which includes both the delirium and mania, was first introduced by Kraepelin in 1921, described by Calmeli, and Luther Bell provided the first systematic description (Bell's Mania) [1] to describe a syndrome characterized by acute onset of excitement, grandiosity, emotional lability, disorientation, and altered consciousness, which are typical symptoms of mania and delirium [2]. Individuals experiencing this condition often have poor or absent recall of the events that occurred during the episode. Taylor and Fink have classified it as a variant of catatonia, while Klerkman considers it to be a variant of classical bipolar disorder [1]. There is ongoing debate about the appropriate nomenclature, with terms such as “lethal catatonia” and “malignant catatonia” proposed alongside delirious mania. Delirious mania, at present, resembles the diagnostic ambiguity like that of catatonia few decades ago, falling in the catatonic spectrum or bipolar spectrum [2]. It is considered to be an uncommon syndrome, but it may be under-recognized as some studies have suggested that 15%–20% of acutely manic patients exhibit signs of delirium [3]. It is not included in the classification of Diagnostic and Statistical Manual of Mental Disorders 5th Edition (DSM-5) or International Classification of Diseases 11th Revision (ICD-11).

The pathophysiology behind delirious mania is unknown; possible mechanism could be a state of decreased acetylcholine (primary neurotransmitter of the ascending reticular activating system) responsible for reduced alertness and inattention, and increased dopamine activity leading to perceptual disturbances and paranoia that are often associated with delirium and mania [4].

Although there are no standardized diagnostic guidelines available for delirious mania, the simultaneous presence of delirium and manic symptoms serves as a clinical indicator. Several rating scales can be potentially helpful in evaluating this condition, including the Delirium Rating Scale-Revised-98 (DRS-R-98) for delirium, the Young Mania Rating Scale (YMRS) for rating manic symptoms, the Bush-Francis Catatonia Rating Scale (BFCRS) for catatonia [1]. The purpose of this case report is to highlight the need and difficulty to identify and adequately address delirious mania. Failure to identify can lead to a rapid escalation of symptoms and pose a life-threatening risk, high morbidity and mortality. There is no consensus in the diagnosis and treatment of delirious mania. Different authors advocate treating differently, considering it as catatonia or mood disorder, with benzodiazepine and electroconvulsive therapy (ECT) or mood stabilizers and antipsychotics, respectively [5, 6]. We present an old lady in her early 80s, a case of bipolar affective disorder with possible delirious mania.

## 2. Case Report

An 82-year old married female from Hindu nuclear family with history of Alzheimer's dementia in first degree relative, was treated for depression 9 months prior presentation on the outpatient basis. She remitted after 2 months and antidepressant was continued for 7 months, till 1 month prior to this episode. There was no history of forgetfulness or other features of dementia, substance use or any chronic medical illness. She presented with persistent elevated mood, marked feeling of well-being, talkativeness, overspending, increased appetite, increased energy, and overactivity for 10 days. In her 9th decade, she would walk briskly to a temple at the top of a mountain which was not her usual self. She was not aggressive or hostile. Four days prior admission, she was found to be confused, not recognizing place and person, and was unaware of time. She stopped self-care, would pass urine and stool in places other than the toilet, would not drink water saying it was mixed with poison and mumble to herself. She was unable to concentrate, was restless, and would not sleep at night. She was aggressive at times, ran out of the house naked, and had to be physically restrained. When brought to hospital, she was agitated, incoherent, and had to be kept in 4-point restraint. There was stupor and echolalia but no other signs of catatonia. The physical examination was within normal limits except tachycardia. There was spontaneous movement and pupils were normal but other neurological examination could not be completed due to uncooperativeness and agitation. On mental state examination, orientation was impaired to time, place, and person, attention and concentration could be aroused but ill-sustained, speech rate was increased with flight of ideas, affect was irritable, and delusion of grandiosity was present. The investigations including basic blood parameters, urine routine examination and culture, thyroid function test, calcium, albumin, magnesium, vitamin-B12, and vitamin-D were within normal range. CT-head was normal except mild cortical atrophy. The DRS-R-98 score was 20 with YMRS score of 22 and BFCRS score of 5. She was started on tablet Quetiapine and optimized to 400 mg with injection Haloperidol 2.5-mg SOS. There was minimal improvement till 5th day, so tablet Divalproex 500 mg was added and optimized to 1,000 mg. ECT was kept in consideration but there was improvement in delirium from the 8th day. Manic symptoms like overfamiliarity, talkativeness, and euphoria remained for the next 7 days. She was discharged after 3/4th improvement in manic symptoms after 24 days of hospital admission with YMRS score of 8. She was compliant to medication and was maintaining well till 3 months of follow-up.

## 3. Discussion

This case report describes an elderly female patient with a history of depression, who presented with manic symptoms and delirium. The patient exhibited symptoms such as persistent elevated mood, increased energy, overactivity, talkativeness, and overspending. However, after 6 days, she became confused, disoriented, and exhibited altered behavior, including aggression and poor self-care.

The presentation of this patient as delirious mania has been very typical as described by Bond [6], except for age. The patient had acute onset of manic and delirious symptoms on the background of mood disorder. Delirious mania is considered to be an uncommon condition, but it may be under-recognized, and studies have suggested that a significant percentage of acutely manic patients exhibit signs of delirium [2, 4].

The lack of standardized diagnostic guidelines for delirious mania poses a challenge in identifying and addressing this condition. However, the simultaneous presence of delirium and manic symptoms can serve as a clinical indicator. The history, examination, investigations, and use of objective scales like DRS-R-98 to recognize delirium, YMRS to assess manic symptoms, and BFCRS to exclude catatonia, helped to overcome the diagnostic challenges.

Fink [2] explained the difficulty to distinguish delirious mania from excited or malignant catatonia. Malignant catatonia is severe form of delirious mania characterized by sign-symptoms of catatonia with motoric agitation, hyperthermia, and may progress to coma and death, which was not present in the patient [7]. As she was elderly, delirium superimposed on dementia was one of our differentials but there were no features of dementia. Then we focused on delirious mania and analyzed the need of ECT along with medications. The effective treatment comprises of the combination of lithium and neuroleptic medications and ECT is considered as a safe and effective alternative [2]. Different other studies have suggested that the definitive treatment for delirious mania is ECT and high-dose benzodiazepines [5, 8]. We refrained from using benzodiazepines in view of increased risk of delirium and paradoxical agitation in elderly [9]. Delirium demanded ruling out of physical illness, for which we ran different tests, which were within normal limits. There were no core features of catatonia, so pharmacotherapy was optimized, which showed excellent response within a week, with the combination of second generation antipsychotic and mood stabilizer.

We could not find the consensus or definite guidelines in the management of delirious mania. Overall, this case report highlights the challenges in diagnosing and managing delirious mania, the importance of promptly recognizing and treating the condition, and the need for further research to establish clear diagnostic criteria and treatment guidelines.

## Data Availability

No underlying data were collected or produced in this study.
